# Maternal peripartum stress: A new consensus-based definition

**DOI:** 10.1007/s00737-026-01741-y

**Published:** 2026-07-01

**Authors:** Déborah Fort, Susan Ayers, Danny Horesh, Susan Garthus-Niegel, Anna-Lena Zietlow, Jonathan Handelzalts, Rafael A. Caparros-Gonzalez, Fiona Alderdice, Valentine Rattaz, Antje Horsch, WG 1.1 COST Action 22114

**Affiliations:** 1 Institute of Higher Education and Research in Healthcare, Faculty of Biology and Medicine, University of Lausanne, Lausanne, Switzerland; 2 City St George’s, University of London, London, United Kingdom; 3 Department of Psychology, Bar-Ilan University, Ramat Gan, Israel; 4 Department of Psychiatry, New York University, New York, United States; 5 Institute and Policlinic of Occupational and Social Medicine, TU Dresden, Dresden, Germany; 6 Institute for Systems Medicine (ISM), Faculty of Medicine, Medical School Hamburg, Hamburg, Germany; 7 Faculty of Psychology, Clinical Child and Adolescent Psychology, TU Dresden, Dresden, Germany; 8 School of Behavioral Sciences, Academic College of Tel Aviv-Yafo, Tel Aviv, Israel; 9 Department of Psychiatry, University of Michigan–Ann Arbor, Ann Arbor, United States; 10 Department of Nursing, Faculty of Health Sciences, University of Granada, Granada, Spain; 11 Instituto de Investigacion Biosanitaria ibs.GRANADA, Granada, Spain; 12 National Perinatal Epidemiology Unit, Nuffield Department of Population Health, University of Oxford, Oxford, United Kingdom; 13 School of Nursing and Midwifery, Queen’s University Belfast, Belfast, United Kingdom; 14 Center for Family Studies, Department of Psychiatry, University Hospital of Lausanne, Lausanne, Switzerland; 15 Faculty of Psychology and Educational Sciences, University of Geneva, Geneva, Switzerland; 16 Neonatology Service, Department Woman-Mother-Child, University Hospital of Lausanne, Lausanne, Switzerland; 17 COST Action CA22114, European Cooperation in Science and Technology, Brussels, Belgium

**Keywords:** Maternal, Peripartum stressor, Peripartum stress appraisal, Peripartum stress response, Peripartum stress definition

## Abstract

The peripartum period is a time of increased vulnerability to stress for women, yet no consensus definition of maternal peripartum stress exists. This paper reports work from European COST Action 22114 (TREASURE), which identified key concepts related to maternal peripartum stress in the literature and developed a consensus definition via a qualitative Delphi process involving 161 international researchers and clinicians. The resulting definition supports a shared understanding, enabling more targeted research and evidence-based clinical practice to improve perinatal care.

## Introduction

The peripartum period, defined here as conception to the end of the first postpartum year, represents a critical developmental transition, often accompanied by increased perceived stress and heightened stress responses in (expectant) mothers. Maternal peripartum stress responses have been consistently associated with adverse outcomes for the entire family (including the infant, the co-parent, and sibling(s)), including increased risk of preterm birth, reduced breastfeeding, impaired mother–infant bonding, and alterations in child developmental outcomes and health (Oyetunji & Chandra 2020).

Research into problems with maternal adjustment in the peripartum period has predominantly focused on depression and anxiety, with the term “peripartum stress” often used interchangeably with anxiety or distress. Numerous studies, as well as theoretical models, have shown that stress is distinguishable from other manifestations of psychopathology, on both the psychological and neurobiological levels. However, theories of stress and empirical evidence suggest that depression, anxiety, and stress are distinct dimensions, which may contribute differently to maternal peripartum adjustment (Liou et al. 2014). For example, there is longitudinal evidence that maternal stress and anxiety follow different trajectories from pregnancy to 4–6 weeks postpartum in community/low risk samples. Maternal peripartum stress may thus constitute a distinct process. Indeed, some evidence shows that stressors specifically linked to pregnancy, such as pregnancy-related bodily changes, may be more important in predicting birth outcomes (e.g., preterm delivery or low birthweight), than general stressors (Lobel et al. 2008). However, there remains no clear consensus on how to conceptualize maternal stress specific to the peripartum period. This lack of consensus and clear conceptualization hinders the development of understanding, as well as targeted research and clinical applications. Establishing this definition is therefore a critical step towards a shared understanding for research and evidence-based clinical practice that benefits mothers and their families.

### Identifying key concepts related to maternal peripartum stress

The work reported here was carried out as part of the European Co-operation in Science and Technology (COST) Action 22114 - Maternal Perinatal Stress and Adverse Outcomes in the Offspring: Maximising infants´ development (TREASURE), a multidisciplinary international research network. Conceptual groundwork was identified from articles included in a pre-registered, comprehensive scoping review of reviews aimed at mapping maternal peripartum stressors and stress responses and relevant conceptualizations of maternal peripartum stress (for further details see Fort, Rattaz (2024)). 

We identified three key concepts of maternal peripartum stress: peripartum stressors, appraisals, and stress responses. Lobel (1994) observed that studies using a multidimensional assessment of prenatal maternal stress, capturing stressors, appraisals, and responses, provided the most consistent and persuasive evidence linking stress to adverse birth outcomes. Lazarus and Folkman (1984) defined a stressor as an external or internal event that has the potential to tax or exceed an individual’s resources. Here we propose that peripartum stressors are circumstances specifically linked to the peripartum period. Examples of prepartum stressors are multiple gestation pregnancies, hypertensive disorders of pregnancy, gestational diabetes, pre-eclampsia, and hyperemesis gravidarum. Placenta praevia and postpartum haemorrhage are examples of childbirth-related stressors. Examples of postpartum stressors are maternal sleep disturbances. Peripartum maternal stressors may also include infant-related factors, such as low birthweight or admission to the neonatal intensive care unit. Such peripartum stressors would not have occurred if the woman had not been pregnant, had not given birth, or was not in the first year postpartum. This is in contrast to general stressors that may occur during the same period in life (e.g., major illness of a loved one). Of note, specific physiological changes that occur during the peripartum period may also constitute stressors (e.g., increased blood pressure linked to the pregnancy can be associated with altered hypothalamic–pituitary–adrenal axis activity and cortisol levels) (Schowe et al. 2024).

We propose that peripartum appraisals will take place following the occurrence of a peripartum stressor and/or a general stressor that could affect the physical/mental integrity of the mother and/or her (future) child. Appraisal was defined by Lazarus and Folkman as the cognitive evaluation of a situation’s relevance to an individual’s well-being and its potential impact (Lazarus & Folkman 1984). This process entails judging whether the situation constitutes a threat, challenge, or harm/loss, and evaluating the individual’s perceived resources and capacity to cope. Appraisals will consider the perceived individual, social, and economic resources available to manage the stressor. Mothers will evaluate whether their personal capacities and available resources are sufficient to meet the challenges posed by the stressors. Personal capacities are shaped by parents’ personality traits, coping styles, and strategies, while social resources depend on access to both emotional and instrumental support. Such support may come from couple and family relationships, informal networks of extended family and friends, or formal assistance provided by professional caregivers. Of note, during the peripartum period, the neurocognitive processes underlying stress appraisals are modulated by peripartum-specific physiological adaptations central to salience detection, threat evaluation, and emotion regulation (Cardenas et al. 2020). This in turn is likely to influence one’s perceived capacity to cope.

The outcome of the appraisal process is a stress response (i.e., physiological, emotional, and behavioural reactions), described by Selye (1936) as the body’s response to any demand. It represents a dynamic interaction between the individual and the environment over time, intrinsically linked to the capacity for adaptation and regulation in support of the organism’s balance and wellbeing (Cardwell 2013). The stress response is influenced by individual and cultural factors, which may interact with comorbid mental health conditions that tend to peak during the peripartum period. Furthermore, the peripartum stress responses also involve physiological, emotional, cognitive, and behavioural reactions, which may be modulated by peripartum physiological changes as well as comorbid mental health conditions occurring during the peripartum period, such as peripartum depression or anxiety disorders, childbirth-related posttraumatic stress disorder, or postpartum psychosis (Howard & Khalifeh 2020). This stress response may have short and long-term consequences for both the mother and her infant, as well as the co-parent and sibling(s). In addition, behavioral alterations are associated with stress-related psychopathology, such as impaired parental bonding, sensitivity, or increased family conflict, which in turn shape the infant’s early environment and developmental trajectory (Howard & Khalifeh 2020).

These concepts extracted from our scoping review were used to draft a definition of peripartum stress, which was subsequently refined through a qualitative Delphi method (Sekayi & Kennedy 2017). This process was conducted between March and June 2025 with n = 161 multidisciplinary (e.g., 41.6% psychology, 18% midwifery, 16.1% nursing, 11.2% obstetrics/gynaecology, 8.7% biology, 5.6% psychiatry, etc.), international researchers (59%) and/or clinicians (37.3% and 3.7%, respectively), and representatives of a United-Kingdom-based service user association (*Make Birth Better*). In each round (two face-to-face and two online), a draft definition was presented on which participants provided narrative comments to guide revisions. Unanimous agreement was reached at the fourth round and resulted in the following definition:

“Maternal peripartum stress refers to the combination of stressors, appraisals, and stress responses associated with the peripartum period. The peripartum period is considered a major life transition and a time of significant physical, psychological, social, and economic changes for expectant and birthing mothers, during which they may be exposed to several stressful situations, including peripartum-specific changes, complications, or peripartum context-specific stressors (peripartum stressors) in addition to general stressors. When mothers experience these stressors as exceeding their personal and/or social resources and/or coping mechanisms (appraisals), a maternal peripartum stress response may result. This response may be reflected by consequences, including emotional, cognitive, behavioural, social, economic, and physical changes in the short and/or longer term.”

**Fig. 1:**
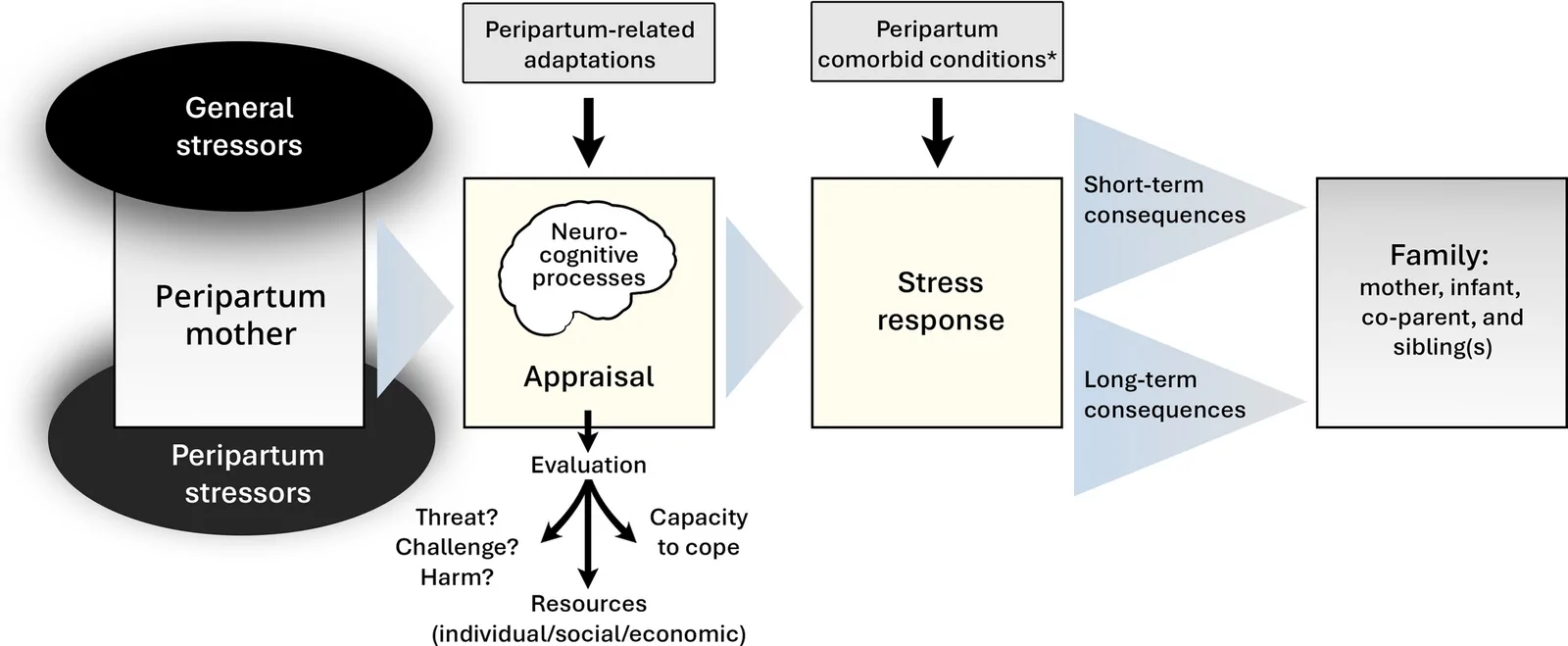
Multidimensional conceptual framework of maternal peripartum stress. *Note*. *Peripartum comorbid conditions are co-occurring physical and mental health conditions, such as anxiety, depression, hypertensive disorders, or pre-eclampsia

This definition, graphically depicted in Fig. 1, aims to provide a common understanding and conceptualisation of maternal peripartum stress, which does not consider specificities of particular groups of individuals, such as adoptive parents or parents who experienced perinatal loss. However, this definition can be adapted if necessary, including in relation to different cultural contexts and perinatal at-risk groups. Furthermore, although the definition focuses solely on expectant and birthing mothers, it has the potential to be adapted to other parents following further research.

### Discussion and future directions

Having a consensus-based definition provides the basis for a common understanding of peripartum stressors, appraisals, and stress responses, which is key for stimulating focused and relevant research and, in turn, evidence-based clinical development to ultimately benefit mothers and their families. This new peripartum definition may inform the development of specific instruments for assessing aspects of maternal peripartum stress (i.e., peripartum stressors, appraisals, and stress responses). For example, a checklist may be developed to identify peripartum-specific stressors. Appraisals of peripartum-specific stressors assessing the perception of threat to the mother and/or the (unborn) infant and their resources and coping mechanisms may be evaluated following the development of a dedicated self-report questionnaire. A comprehensive assessment of stress responses should involve the measurement of emotional, cognitive, behavioural, social, economic, and physical changes in the short and/or longer term.

Advancing knowledge in this area constitutes an essential step toward disentangling the complex relationships between (distinct) pathways of peripartum stress, anxiety, and depression and their respective influences on maternal, infant, co-parental, and sibling(s) outcomes. This new definition takes a continuous approach to maternal adjustment to peripartum-specific stressors, resulting in stress responses, which may ultimately but not necessarily lead to pathology (e.g., anxiety or depression or gestational diabetes). Studying the stress response in the peripartum context may help identify markers for this transition from an adaptive stress response to pathology. Clinically, identifying peripartum stressors will facilitate the early detection of individuals at risk for adverse outcomes (e.g., preterm birth; Lobel et al. 2008) and guide the design of targeted interventions to prevent the negative consequences of maternal peripartum stress.

## Data Availability

No datasets were generated or analysed during the current study.
